# Supplementary material for: Content-rich biological network constructed by mining PubMed abstracts
Source: BMC Bioinformatics. 2004 Oct 8;5:147. doi: 10.1186/1471-2105-5-147 (PMC528731; doi:10.1186/1471-2105-5-147)
Supplement: Additional File 2 — The original results of the above study (non-essential files are deleted to keep the file size under the limit set by BMC bioinformatics). [file 1471-2105-5-147-S2.bz2 › chilibotAdditionalFile2/dip20/232ID7931156E751/html/UL9_UL8.html]

 


 **UL9** and **UL8** 
  
Found 20 abstracts in PubMed, retrieved 20.  
 

 What does Google say? 
 PDF only 
| .edu only 

---

**Interactive relationship** (e.g. stimulation, inhibition, etc)

**Neutral relationship**- Similar experiments using a recombinant virus that expressed the HSV 1 origin binding protein OBP  [ **UL9** ] ,  **UL9** , demonstrated a direct physical interaction between the helicase primase complex and OBP  [ **UL9** ]  which involved the  **UL8**  subunit.  Ref: 7931156 J Gen Virol, 1994
- The 5 end of the UL10 mRNA, which is transcribed from the strand opposite that specifying the  **UL8** ,  **UL8** .5,  **UL9** , and  **UL9** .5 transcripts, lies within the  **UL9**  open reading frame at nucleotide 2 44, while the 3 terminus was mapped to nucleotide 2 66.  Ref: 8207800 J Virol, 1994
- Analysis of transcripts and time course studies demonstrated that the  **UL8**  protein is translated from a delayed early expressed 3.1 kb polyadenylated mRNA which initiates within the  **UL9**  ORF.  Ref: 9460934 J Gen Virol, 1998
- The C terminal DNA binding domain of OBP  [ **UL9** ]  is dispensable for this interaction, as evidenced by the ability of MAb 0811 to co precipitate a truncated  **UL9**  protein, containing only the N terminal 535 amino acids, with  **UL8** .  Ref: 7931156 J Gen Virol, 1994
- A truncated protein, consisting of the C terminal 487 amino acids of OBP  [ **UL9** ] , termed OBPC, is the product of the HSV  **UL8** .5 gene and binds to origin sequences, although OBPC s role in HSV DNA replication is not yet clear.  Ref: 11435559 J Virol, 2001
- Examination of the nucleotide sequence of the  **UL8** .5 transcript revealed a potential open reading frame that overlaps and is in frame with the C terminal half of the open reading frame encoding the origin binding protein OBP  [ **UL9** ] , the product of the  **UL9**  gene.  Ref: 8207800 J Virol, 1994

**Non-interactive relationship** (e.g. studied together, co-existance, homology, etc.)

- On the basis of these results, we present a model for prereplicative site formation in infected cells in which the helicase primase components UL5,  **UL8** , and UL52, the origin binding protein  **UL9** , and the viral single stranded DNA binding protein ICP8 assemble together to initiate the process.  Ref: 8627698 J Virol, 1996
- Transcriptional analysis of the region of the herpes simplex virus type 1 genome containing the  **UL8** ,  **UL9** , and UL10 genes and identification of a novel delayed early gene product, OBPC.  Ref: 8207800 J Virol, 1994
- Since this pattern can form in cells infected with mutants which are defective in UL5,  **UL8** ,  **UL9** , or UL52 in the presence of polymerase inhibitors C.  Ref: 9151871 J Virol, 1997
- The region of the UL component of the herpes simplex virus type 1 genome between nucleotides 1 93 and 2 50 includes three open reading frames that code for the protein products of the  **UL8** ,  **UL9** , and UL10 genes D.J.  Ref: 8207800 J Virol, 1994
- The 5 ends of the  **UL8** ,  **UL8** .5, and  **UL9**  transcripts were mapped to nucleotides 2 8 51, and 2 81, respectively.  Ref: 8207800 J Virol, 1994
- To improve our ability to study these proteins, we have expressed UL5,  **UL8** ,  **UL9** , and UL52 in insect cells by using the baculovirus expression system.  Ref: 2535726 J Virol, 1989
- Time course studies demonstrated that the  **UL8**  and  **UL9**  transcripts are members of the early kinetic class, the  **UL8** .5 mRNA is a delayed early transcript, and the  **UL9** .5 and UL10 transcripts belong to the true late kinetic class.  Ref: 8207800 J Virol, 1994
- Within this region eight open reading frames were identified whose deduced amino acid sequences exhibited homology to the UL6, UL7,  **UL8** ,  **UL8** .5,  **UL9** , UL10, UL11, and UL12 protein products of herpes simplex virus type 1.  Ref: 9155870 Arch Virol, 1997
- These studies reveal that in the absence of ICP27, expression of the UL5,  **UL8** , UL52,  **UL9** , UL42, and UL30 genes is significantly reduced at the level of mRNA accumulation.  Ref: 8627723 J Virol, 1996
- The 3 ends of the  **UL8** ,  **UL8** .5,  **UL9** , and  **UL9** .5 transcripts are coterminal at nucleotide 1 97.  Ref: 8207800 J Virol, 1994
- These genes were designated UL5,  **UL8** ,  **UL9** , UL42, and UL52 and were predicted to encode proteins with molecular weights of, respectively, 9 0 0 0 00, and 11 00.  Ref: 2826807 J Virol, 1988
- We constructed recombinant viruses based on the herpes simplex virus type 1 mutant tsK which individually were able to express the products of four viral DNA replication genes UL5,  **UL8** ,  **UL9**  and UL52 in the absence of any of the other proteins required for viral DNA synthesis.  Ref: 1312116 J Gen Virol, 1992
- We have mapped and characterized the overlapping transcripts in this region and have found that, in addition to the low abundance  **UL8**  and  **UL9**  transcripts and the abundant UL10 transcript, at least two additional transcription units, designated  **UL8** .5 and  **UL9** .5, are specified by this region of the genome.  Ref: 8207800 J Virol, 1994
- The results shown in this paper are consistent with the model that the five proteins, ICP8, UL5,  **UL8** ,  **UL9** , and UL52, form a scaffold.  Ref: 12634381 J Virol, 2003
- Transcriptional analyses indicated presence of 3 coterminal mRNAs for genes  **UL8** ,  **UL8** .5, and  **UL9**  as well as for genes UL6 and UL7, respectively, while UL10 was represented by a very abundant unique transcript.  Ref: 9155870 Arch Virol, 1997
- Cotransfection of the cloned, replication competent AAV 2 genome together with the seven HSV replication genes UL5,  **UL8** ,  **UL9** , UL29, UL30, UL42, and UL52 led to productive AAV replication.  Ref: 1850024 J Virol, 1991
- The ordered assembly of the herpes simplex virus HSV type 1 replication apparatus leading to replication compartments likely involves the initial assembly of five viral replication proteins, ICP8,  **UL9** , and the heterotrimeric helicase primase complex UL5  **UL8**  UL52, into replication foci.  Ref: 12634381 J Virol, 2003
- By the use of HSV mutants for the  **UL9**  gene we show here that HSV can induce DNA amplification in the absence of lytic viral growth in contrast to replication negative mutants for either the  **UL8**  or UL52 gene used as control.  Ref: 2171219 Virology, 1990
- The assembly of complete HSV replication compartments and incorporation of BrdU were both abolished by treatment with phosphonoacetic acid and by omission of any one of the seven viral replication proteins, UL5,  **UL8** ,  **UL9** , UL42, UL52, SSB, and Pol, that are essential for viral DNA replication.  Ref: 9060678 J Virol, 1997
- The observed effect on herpes simplex virus DNA replication was not caused by a decreased transcription of replication genes inasmuch as the levels of  **UL8** ,  **UL9** , UL29, and UL30 rmRNAs were unaffected by the drug.  Ref: 8676478 J Virol, 1996
- Various immunological assays suggest that four of these proteins UL5,  **UL8** ,  **UL9** , and UL52 are made in infected cells in very low abundance relative to the other three.  Ref: 2535726 J Virol, 1989
- Homologs of essential HSV 1 replication proteins,  **UL8**  and  **UL9** , were also identified.  Ref: 9155884 Arch Virol, 1997
- We observed that four replication proteins, UL5,  **UL8**  UL52, and  **UL9** , are necessary for the localization of ICP8 UL29 to prereplicative sites natural infection conditions.  Ref: 8627698 J Virol, 1996
